# Study of LZ-Based Location Prediction and Its Application to Transportation Recommender Systems

**DOI:** 10.3390/s120607496

**Published:** 2012-06-04

**Authors:** Alicia Rodriguez-Carrion, Carlos Garcia-Rubio, Celeste Campo, Alberto Cortés-Martín, Estrella Garcia-Lozano, Patricia Noriega-Vivas

**Affiliations:** Department of Telematic Engineering, University Carlos III of Madrid, Avda. de la Universidad 30, 28911, Leganés, Madrid, Spain; E-Mails: cgr@it.uc3m.es (C.G.-R.); celeste@it.uc3m.es (C.C.); alcortes@it.uc3m.es (A.C.-M.); emglozan@it.uc3m.es (E.G.-L.); pnoriega@it.uc3m.es (P.N.-V.)

**Keywords:** GSM-based location, prediction, LZ, LeZi Update, Active LeZi, recommender system, ambient intelligence, ubiquitous computing

## Abstract

Predicting users' next location allows to anticipate their future context, thus providing additional time to be ready for that context and react consequently. This work is focused on a set of LZ-based algorithms (LZ, LeZi Update and Active LeZi) capable of learning mobility patterns and estimating the next location with low resource needs, which makes it possible to execute them on mobile devices. The original algorithms have been divided into two phases, thus being possible to mix them and check which combination is the best one to obtain better prediction accuracy or lower resource consumption. To make such comparisons, a set of GSM-based mobility traces of 95 different users is considered. Finally, a prototype for mobile devices that integrates the predictors in a public transportation recommender system is described in order to show an example of how to take advantage of location prediction in an ubiquitous computing environment.

## 1. Introduction

Many applications are based on users' current context, but sometimes this is not enough. If the application reacts when the current context changes and the task to carry out due to the change takes some time to complete, then the result of this reaction may not come in time. Even more, reacting too late to context changes may worsen the user experience by showing information out of context or performing unexpected actions for the user. Anticipation and improvement of user experience are some of the key features needed by Ambient Intelligence technologies in order to bring this intelligence to the environments and make them sensitive to users [1–3]. To achieve these features we could add information about the most probable next context, so that those applications would have more time to make the necessary adjustments and be ready when the users' context actually changes. This work is focused on a particular aspect of the context, the users' location, thus aiming to offer services based on users' future destinations. More precisely, we are going to study some tools for estimating those future locations: the so-called location prediction algorithms.

Location predictions may be an interesting improvement for ubiquitous computing applications, such as Location Based Services (LBSs). The prediction of users' next location would allow to provide services related not only to their current location, but also to their future destinations. This way each user could be aware of information related a certain place (restaurant, museum) and decide whether to stop by that place or not right before getting there. The mobile phone itself may also be aware of users' future location, thus being able interact with that location (e.g., an office or home) so it is prepared somehow when the user gets there (computer, lights or heat turned on). Ambient Intelligence applications can also exploit the movement patterns learned and predicted, for example for anomaly detection in elderly people care systems to determine if they get lost [4].

Some authors propose to calculate the predictions in the network [5,6]. However, in this case we are interested in learning and predicting using the mobile terminal itself because of several reasons, namely: (i) the advantages drawn from the fact that each user (terminal) learns and predicts her location, thus making the process distributed (with respect to the option of the network doing all the work); (ii) the improvement in privacy, since there is no need for sending location data through the network (the device obtains that information and process it); and (iii) the possibility of choosing the preferred technology for location tracking among the many ones integrated in mobile devices (GPS, WiFi or GSM/UMTS).

However, making any kind of data processing in mobile devices is tied to a concern on the limited memory and processing speed. Therefore, the selection of the prediction algorithm to use needs to take into account such restriction. There are several methods that do not require complex computation, such as Markov models, Bayesian networks and text compression-based techniques. Taking into account some of the results of Bhattacharya and Das work [7], the text compression algorithms represented by LZ family outperforms theoretically a Markov model of any order. Besides, LZ algorithms do not need training phases (but Bayesian networks do) and are thus able to adapt to routine changes in real time, which is an interesting feature regarding the variability of users' behavior. Therefore, we have centered our research focus on this promising family, comprised by three algorithms: LZ [8], LeZi Update [7] and Active LeZi [9].

One of the main contributions of our research is the new approach followed when analyzing these algorithms. Instead of considering them as a block process, we split each one into two independent phases: tree updating scheme and probability calculation method. This approach allows to study which instance of each phase is the best for reducing error rate and achieving the lowest resource consumption. We discuss the working principles of these predictors and how to make this separation in Section 2.

In Section 3 we present the results obtained after evaluating the combination of different instances of each phase, regarding both error rates and resource consumption. The analysis is based on GSM location records, but there are similar analyses using Wi-Fi data [10]. In a previous study [11] we shown preliminary results obtained after processing 10 mobility traces randomly chosen from a set of 95 users. The contributions of the current work over [11] are: (i) the analysis of the results obtained after processing of complete users' traces set using the prediction algorithms, so as to validate the performance evaluation results shown in the previous work; (ii) the analysis of the results drawn from processing some mobility traces we have recorded for comparing them with those of the anonymous users; (iii) the explanation of certain unexpected results related to Active LeZi algorithm; and (iv) the description of a prototype developed in order to check how the algorithms work when they are integrated in a more complex application. The prototype, described in Section 4, aims to recommend the bus line that best matches the path the user seems to be covering, according to the predictions made by the LZ algorithms.

To finish the paper we summarize the main conclusions along with some future research lines in Section 5.

## 2. Location Prediction Algorithms

As stated in [1], among the AmI technologies that provide responsiveness and adaptation to the environment, we can find different types of reasoning method, namely user modeling, prediction and recognition, decision making and spatial-temporal reasoning. The two first ones, modeling and prediction, applied to mobility scenarios, are performed by location prediction algorithms.

There exists a wide variety of this kind of algorithms. Some of them use Global Positioning System (GPS) coordinates as input information [12–14], whilst some others deal with symbolic representation of places [15] or regions. This last case refers to the use of WiFi access points (APs) [10,16] or GSM/UMTS base stations (BS) identifiers [5,17] that can be used to represent the coverage area under that AP or BS. The location technology used has effects on the location accuracy as well as on the location information retrieval process. GPS allows to obtain the location with an error lower than 50 m [18], but its use entails two main problems. The first one is related to coverage, since there is no GPS coverage inside buildings, where users spend most of their daily lives. The second problem derived from using GPS is its high energy consumption, which drains the terminal battery really fast. Although the accuracy of WiFi system (lower than 100 m, *i.e.*, the AP coverage area [19]) and GSM/UMTS network (from 100 m in urban areas to several kilometers in rural areas [20]) are worse than that of GPS, both of them avoid the problems exposed above. Since GSM/UMTS network has global coverage, we will be using this technology in order to locate users.

We can also classify location prediction algorithms depending on where the prediction is made. Some works [5,6] propose all the processing to be made by the system or the network, so that the terminal is not involved in the process. However, this approach has several drawbacks, as pointed out in [13]. Tracking mobility from the network means to have a central element where all the tracking information will be stored. If this database fails or is compromised, all the data would be lost, or even worse, very sensitive personal information would be disclosed. Moreover, if the terminal needs these location data, some kind of connection must be used in order to retrieve such information. However, it could be the case of needing this information during offline situations. Therefore, many approaches have switched to make the prediction on the terminal itself [13,21]. Nevertheless, it must be noticed that this option has also a drawback. Mobile devices have limited resources, both memory and CPU capacity, for executing algorithms. Thus, it is not possible to use very complex algorithms that take very long time to obtain a prediction. Otherwise, the location forecast would be available too late, when it may not be meaningful for the application anymore.

Focusing on the concrete type of pattern learning and prediction calculation methods, the options are varied, starting from the use of well-known mobility models [6,22] to machine learning methods, both supervised (Bayesian approaches [23,24], neural networks [23,25], Hidden Markov models [26]) and unsupervised (clustering techniques [27], Self-Organazing Maps [24], Adaptive Resonance Theory [28]), or information theory techniques (Markov models [13,14,29], compression algorithms [7–9,15]) among many others.

From this extensive collection of different options, we focus on making both the movement tracking and prediction calculation on the terminal so as to take advantage of distributed computing features, which is in line with AmI principles. Taking into account the limitation imposed by these devices, as mentioned before, we have chosen a family of compression algorithms that can be used for estimating location predictions with low resource needs: the LZ family.

In this section we study the working principles of this family, comprised by three algorithms: LZ, LeZi Update and Active LeZi. They are domain independent, meaning that they consider each location as a different symbol, without taking into account any other information about that location (as opposite to domain dependent algorithms, which make their predictions based on location context information such as coordinates or place function). They work by processing a symbol string, known as movement history or trace (*L*), that represents the locations visited by the user. The predictions made by these algorithms are based on two main hypothesis: (i) user's mobility patterns are repetitive, thus the movement history is a stationary process; and (ii) user's movement follows a probabilistic model, and therefore *L* is also a stochastic process.

There are three main reasons for considering the LZ family algorithms explained in detail along this section:

- They do not need many resources, thus being possible to execute them on mobile devices.
- They take into account changes in user's behavior. Therefore if a user usually visits certain places and at some point starts visiting other locations, the algorithm will realize this change and make the predictions according to the new routine. This is an advantage with respect to the methods that need an initial training phase, such as Bayesian networks, since once the training is done, new routines are not considered because they happened after the parameters of the model were set.
- As stated in [7], these algorithms outperform theoretically a Markov model of any order. LZ algorithms are like Markov models, except for that the order grows dynamically, achieving an optimal value at each step in terms of entropy, *i.e.*, the model encloses the minimum uncertainty with the lowest order (with a higher order model the uncertainty enclosed will be the same), which implies minimum resource consumption.

Along this section we describe the aforementioned algorithms, highlighting the possibility of splitting each one into two independent phases as described at the end of the section.

### 2.1. LZ Algorithm

**Figure 1.**
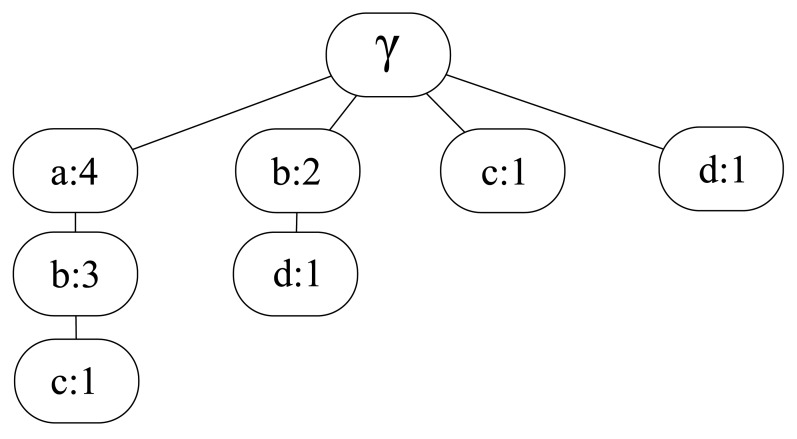
LZ tree after parsing the example movement history *L* = *abababcdcbdab*.

This is the base algorithm and works as follows [8]. Let *γ* be the empty string and *L* the input movement history. LZ algorithm takes *L* and splits it into substrings *s*_0_*s*_1_…*s_m_* such that *s*_0_ = *γ* and for all *j* ≥ 1 the prefix of substring *s_j_* (*i.e.*, all but the last character of *s_j_*) is equal to some previous *s_i_*, for all *i* < *j*. The division is made sequentially, so that when each *s_i_* is parsed, then the algorithm considers only the remaining trace. For example, the movement history *L* = *abababcdcbdab* is divided as follows: *a, b, ab, abc, d, c, bd, ab*. In order to store these substrings (patterns) in an efficient way, LZ algorithm builds the so-called LZ tree, each node of which representing a substring and storing the number of times that substring appears among the parsed patterns. For instance, the tree corresponding to the previous example is the one shown in Figure 1.

After updating the tree, the next step is to calculate the probability for each known symbol to be the corresponding to the next location. In order to do that, LZ algorithm uses an approach proposed by Vitter [30] that can be expressed as in Equation (1):

(1) $$P(X_{n+1}=a|L)=\frac{N^{LZ}(la,L)}{N^{LZ}(l,L)}$$

where *l* is called prediction context and corresponds to the last substring that has been parsed by LZ algorithm; *N^LZ^*(*la, L*) represents the frequency of the substring *la* (*i.e.*, the prediction context followed by symbol *a*) in the LZ tree and *N^LZ^*(*l, L*) represents the frequency of the substring *l* also in the LZ tree. Finally, LZ algorithm chooses the symbol with the highest probability of being the corresponding to the next location.

LZ algorithm has three main drawbacks: (i) patterns between two parsed substrings are lost; (ii) patterns contained within substrings parsed by LZ scheme are also lost; and (iii) Vitter method has problems when a pattern is detected for the first time, since it has not enough information and is not able to make any prediction. The two next algorithms try to overcome these limitations.

### 2.2. LeZi Update Algorithm

Bhattacharya and Das [7] propose to make the same parsing made by LZ algorithm, but instead of adding only the substrings resulting from the LZ parsing, LeZi Update also adds to the so-called LZU tree all the suffixes of each substring. Therefore patterns within substrings are also taken into account. Analyzing the former example, LeZi Update parses *L* as follows: *γ, a, b, ab*{*b*}, *abc*{*bc, c*}, *d, c, bd*{*d*}, *ab*{*b*}, where the substrings outside the brackets correspond to the output of the LZ parsing process, and the ones inside the brackets are the additional ones added by the modifications introduced by LeZi Update algorithm.

Regarding probability calculation method, LeZi Update algorithm uses PPM (Prediction by Partial Matching [31]). This method works as follows. First we need to determine the longest prediction context, *l_k_*, which in this case corresponds to the longest substring (starting by the last symbol of *L*) which is already in the LZU tree (*k* is the length of this context). Then, we have to count the frequency of each substring that has followed this prediction context and the prediction contexts of lower orders (*l_k_*_−1_ up to *l*_0_), as well as what is known as escape event, *esc, i.e.*, the number of times a substring (e.g., *l_k_*) is not followed by any symbol. Besides, LeZi Update applies what is known as exclusion technique [7]. This means that only one-symbol substrings are considered (as we are only interested in predicting the next location), excluding the remaining ones. Once we have this information, the probability of symbol *a* being the next one (*X_n_*_+1_) is calculated as shown in Equation (2):

(2) $$P(X_{n+1}=a)=P_{k}(a)=P(a|l_{k})+P(\text{esc}|l_{k})\cdot P_{k-1}(a)$$

where *P_k_*(*a*|*l_k_*) is the probability of *a* being the next symbol taking into account the prediction context of order *k*; and *P*(*esc*|*l_k_*) is the escape probability when considering the prediction context of order *k* (*i.e.*, the number of escape events following *l_k_*).

This algorithm solves the problems posed by Vitter approach, and the probability estimations are much more complex, taking into account more information.

### 2.3. Active LeZi Algorithm

The algorithm proposed by Gopalratnam [9] is intended to consider the substrings among consecutive parsed patterns when building the so-called ALZ tree, thus solving the remaining problem of LZ algorithm. In order to achieve this, Active LeZi uses a window of variable length, which is determined by the longest pattern parsed by LZ algorithm at each step. Once the length of the window is updated (if needed) and the new symbol is added to it, all the suffixes of the window are added to the tree. In the former example, the window evolution will be as follows: *a, b, a, ab, ba, ab, abc, bcd, cdc, dcb, cbd, bda, dab*, and the ALZ tree includes every suffix of each substring (e.g., for *abc*, suffixes *abc, bc* and *c* are included).

The probability calculation process is based on PPM algorithm, as in the previous case, but this time exclusion method is not applied. This only affects the pattern counting, but the Equation (2) still applies.

With Active LeZi algorithm all the initial problems are solved at the expense of increasing the information stored and therefore the memory and time resources required, as we will see in the next section.

### 2.4. Our Proposal

After describing each algorithm and its working principles, we may realize that they share a common structure. Every algorithm takes each new symbol, processes it to update the corresponding tree and finally calculates some probabilities. Therefore, we can distinguish two stages:

1. Tree updating scheme. It processes each new symbol and updates the corresponding tree which is in charge of storing user's mobility pattern data.
2. Probability calculation method. It uses the updated tree to estimate the probability of each known symbol to be the corresponding to the next location. Once we have all the probabilities calculated, the prediction will be the symbol whose probability is the highest one.

**Figure 2.**
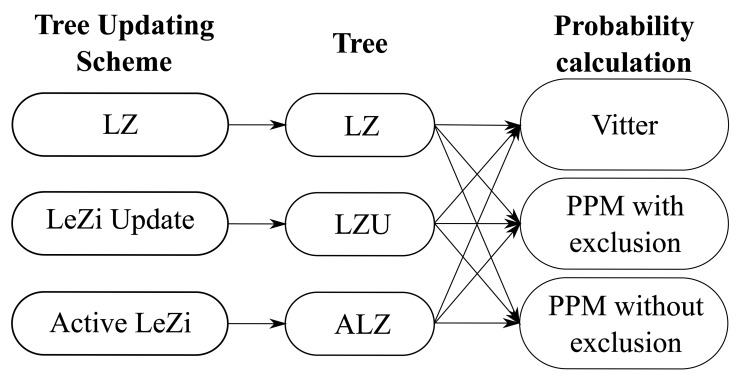
Combinations of the two independent stages.

Figure 2 shows this division and the nine possible combinations. This procedure allows to study each step separately and determine its impact on the performance. Some results derived from processing several traces with these combinations will be shown in the next section.

## 3. Performance Evaluation

In this section we show some results obtained from processing mobility traces with the algorithms described in Section 2. The analysis will be focused on hit rate as well as the memory usage, processing time and power consumption. But before starting with the performance analysis, a description of the mobility data used is provided.

### 3.1. Data Collection Analysis

The set of algorithms explained in Section 2 deals with symbols representing the locations visited by the user. In order to obtain those symbols, two steps are needed. The first step is to gather location-related information, using any of the several technologies integrated in almost every mobile device nowadays that retrieves this information. We have chosen location data based on GSM network information. Devices can record the base station (BS) to which they are connected every time. The BS a user is connected to changes as she moves, and therefore the movement can be followed by tracking the BS series the user has been connected to. During the second step the location information extracted from the GSM network is translated into symbols in the following way. The network splits the space into cells, each one identified by a Location Area Code (LAC) and a cell identifier (CellID). These two parameters can be translated into a unique symbol that represents the zone covered by the cell. Therefore each time the terminal changes from one cell to another, the device records the new location represented by its corresponding symbol.

In order to evaluate the predictors performance we have analyzed two different datasets made up of GSM-based location data. The first one consists of a trace we have recorded, which stores the movements of a person who makes a regular routine during four days, generating a trace that gathers 2,897 cell changes among 33 different cells. The routine starts at user's home, from where she takes a bus to get to her work place, stays there until the evening, when she takes the same bus and makes the round trip to home. However, since we want to study the behavior of the algorithms in general scenarios, we have considered the Reality Mining Project dataset [32], which recorded the movement history of 95 different anonymous users during the 2004–2005 academic year. The traces varies from a few hundreds up to almost 80,000 cell changes, and ranges from less than 100 up to more than 3,000 different visited cells. With the Reality Mining dataset we can analyze the algorithms performance by measuring the average percentage of correct predictions for an extensive number of different users, thus obtaining an idea of the results that can be expected regardless of the mobility characteristics of the specific user. However, we have little information on the movement routines of each particular user in that dataset. Therefore, it is not possible to analyze the temporal evolution of the algorithms' results since we do not have the information on the temporal evolution of the user's movements. In order to fill this gap, we will use the trace recorded by ourselves. With this trace we can observe how a daily routine is mapped to correct predictions, tree size and processing time evolutions.

### 3.2. Hit Rate Analysis

**Figure 3.**
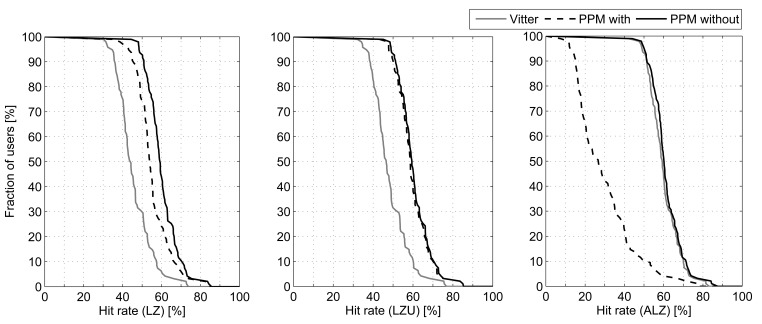
Comparison of hit rate attained when fixing the tree updating scheme and varying the probability calculation method.

We are going to use the 9 combinations shown in Figure 2 to process the traces described above, analyzing each phase individually. Starting with the probability calculation method, Figure 3 represents the percentage of traces (users) that attain, at least, the corresponding averaged hit rate (number of predicted next cell and actual next cell matches divided by the total number of cell changes). For example, in the case of Active LeZi (ALZ) algorithm combined with Vitter or PPM without exclusion (dotted and slashed lines of the rightmost plot), 50% of users achieve, at least, an averaged hit rate around 60%. For this first comparison we have fixed the updating scheme, represented in each of the three subfigures, and applied each probability calculation method. We can see that PPM without exclusion method is the best one in all cases. Regarding the results derived from using Vitter method, we can see that they are very close to those attained by PPM without exclusion, even being a much simpler calculation method, and thus consuming much less resources as we will see later.

**Figure 4.**
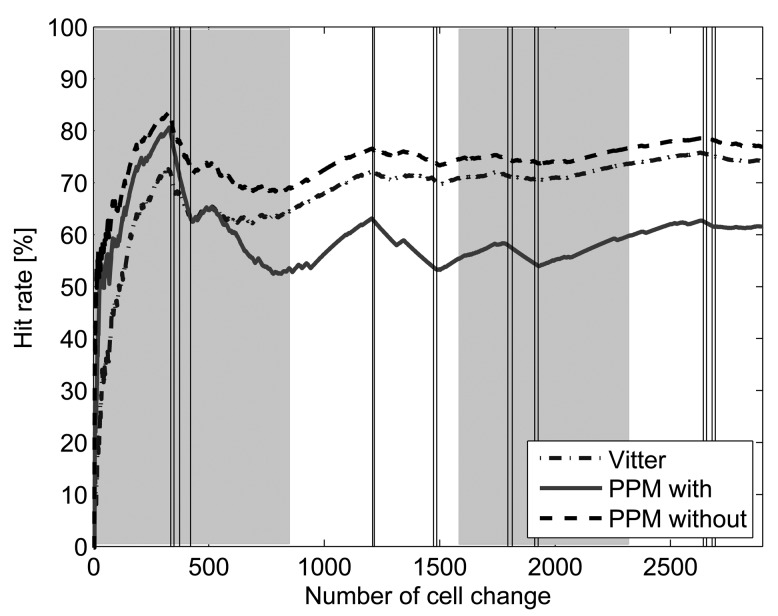
Hit rate evolution when processing the 4 days trace with Active LeZi updating scheme combined with each probability calculation method.

Hit rate achieved by Active LeZi combined with PPM with exclusion method is much lower than in the rest of the cases. This same behavior can be also observed in our 4 days trace. Figure 4 shows the evolution of hit rate along the 4 days, considering Active LeZi updating scheme combined with the three probability calculation methods. As it can be noticed, hit rate achieved by Vitter method is very close to that achieved by PPM without exclusion, whilst PPM with exclusion hit rate remains much lower.

**Figure 5.**
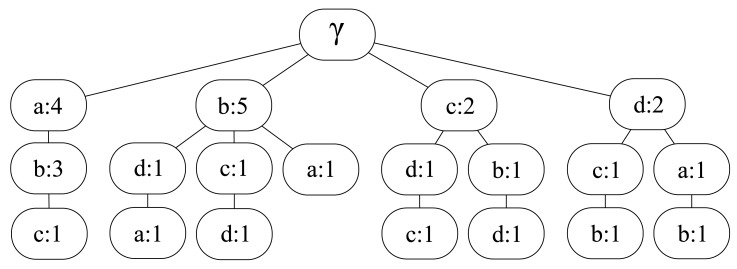
ALZ tree after parsing the example movement history.

This last fact may be surprising, but if we take a deeper look into the working principles of PPM with exclusion method, the reason becomes clear. Let *L* be *abababcdcbdab* and the resulting ALZ tree be the one represented by Figure 5. Table 1 is the table that PPM with exclusion method builds when analyzing that tree, where *l_i_* are the different contexts PPM uses for calculating the next symbol probabilities.

By taking the one-symbol substrings (the ones we are interested in since they represent the next location) we see that most of them have frequency 0 due to the fact that Active LeZi adds to the ALZ tree all the substrings whose length is equal to the window length at each step. Therefore most part of the intermediate tree nodes are only part of longer substrings instead of being a substring by themselves. For example, substring *dcb* has been added in one go and therefore PPM with exclusion method only considers it as a complete substring *dcb*, without noticing about intermediate nodes *dc* or *d*. Therefore, as PPM considers shorter contexts (*l*_1_ = *b, l*_0_ = *γ*), the symbol frequencies are lower (being 0 in many cases) because all or most instances of those nodes are probably part of longer substrings that have been added in one go. This phenomenon entails two conclusions:

- PPM with exclusion does not seem to be very appropriate in our case since we are only interested, by now, in the next event (instead of the next sequence of events).
- The lowest orders are barely taken into account since PPM with exclusion quantifies those frequencies in such a way that they turn to be very low or even 0. This fact is specially critical since the lowest orders are the ones giving rise to better probability estimators as they have the highest number of samples.

**Figure 6.**
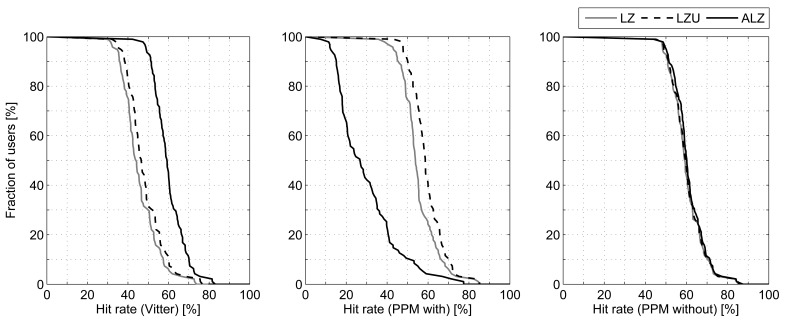
Comparison of hit rate attained when fixing the probability calculation method and varying the tree updating scheme.

With respect to the comparison of updating schemes (*i.e.*, if we fix the probability calculating method and apply different updating schemes), we can see in Figure 6 that Active LeZi (ALZ) is the best choice when working with Vitter and even with PPM without exclusion, although the differences in the last case are very small. LeZi Update works better with PPM with exclusion because of what we have previously discussed.

Without taking into account the combination of Active LeZi with PPM with exclusion method, the results are coherent with those shown in [10]. This conclusion could be foreseen since information gathered by ALZ tree is greater with respect to LZU tree, and the same applies to LZU tree with respect to LZ tree.

Regarding the temporal evolution of the hit rate, Figure 4 shows this evolution for the 4 days trace. Grey and white zones delimit each day. The vertical lines within each day point out when the user leaves home to go to her job, gets there, leaves the work place and arrives home. As it can be observed, the first day the hit rate is very variable since the algorithms are learning the user's routines for the first time. For the rest of the days, the hit rate increases while the user is at home (the only cell changes are due to network issues and usually there are only two or three different cells to which the cell phone switches even when there is no movement associated), whilst the hit rate decreases slightly when the user is away. It has to be taken into account that, although the route between home and work place is always the same, the series of BSs representing that route (the series of BSs the user is attached to during the trip) can be different and are not under the user's control. Therefore, the pattern learned the first day might be different from that of the second day. As time goes by and all possible BSs series representing that route are learned by the algorithms, the hit rate is less affected by these trips, as can be observed during the fourth day.

### 3.3. Resource Consumption

Each of the two phases explained in Section 2.4 has very different effects related to resource consumption. The first one, tree updating scheme, takes care of building the pattern tree thus being tightly coupled with memory consumption because the size of the tree depends on the updating scheme chosen. The second step, which is in charge of calculating probabilities, is related to the processing time and depends on the complexity of the method used.

**Figure 7.**
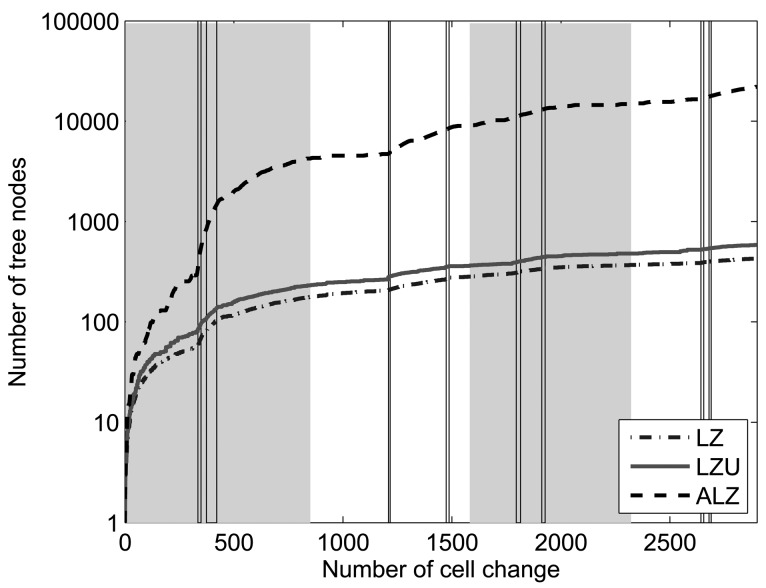
Node count of different trees (log scale).

Figure 7 represents the node count evolution of each tree as each symbol of the 4 days trace is processed. The figure highlights that ALZ tree grows much faster, whereas LZ and LZU tree sizes also start growing quickly but stop increasing so fast at a lower level, achieving a size of two magnitude orders lower than ALZ tree in the end. This result proves our hypothesis that Active LeZi scheme achieves the best hit rate in most cases at the expense of much higher memory consumption, which may be unacceptable for some applications. Regarding the temporal evolution, we can see that the node count increases faster when the hit rate decrease is steeper, which reinforces the idea of the algorithms learning new patterns and thus being unable to make correct predictions.

**Figure 8.**
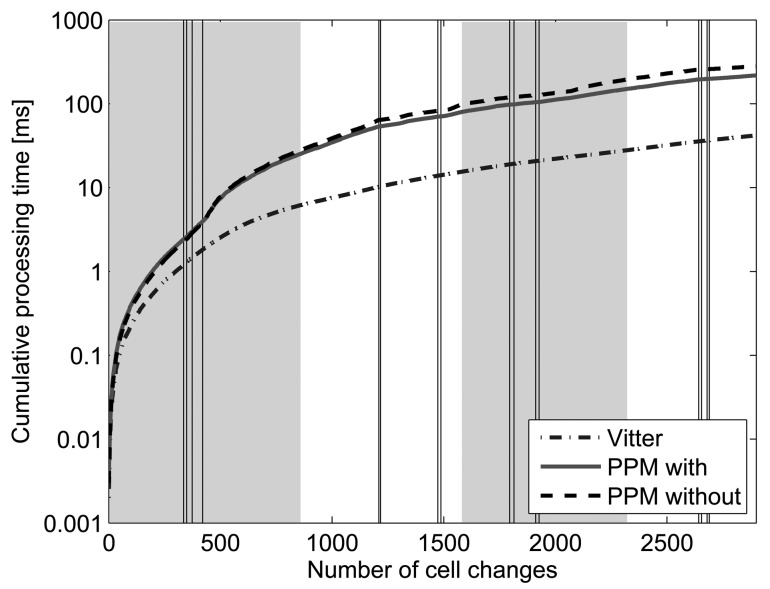
Accumulated processing time needed by Active LeZi updating scheme combined with each probability calculation method (log scale).

With respect to processing time, Figure 8 shows the evolution of the cumulative time spent by Active LeZi combined with each of the probability calculation methods when processing the symbols of the trace analyzed before. It is worth to notice that these are not the times spent for processing each symbol, but each value corresponds to the sum of the previous processing times plus the current one. We have chosen this representation in order to make the comparison among the three probability calculation methods more clear. The instantaneous processing time values are very variable because the prediction task is not the only one executing in the system and the existence of other delay sources makes the processing time a very variable signal. Representing the cumulative value filters the noisy appearance and highlights the differences among the different techniques. These results have been obtained with an Intel Core 2 Duo 2.66 GHz with 4 GB 1,067 MHz DDR3 computer. We can see that whereas Vitter method keeps time processing level very low all the time (it does not achieve 50 milliseconds after processing 2,900 symbols), PPM methods spend a cumulative time around 300 milliseconds in the end, one magnitude order above Vitter case. Therefore, if applications using these algorithms are time sensitive, Vitter method would be more convenient even having lower hit rate.

**Figure 9.**
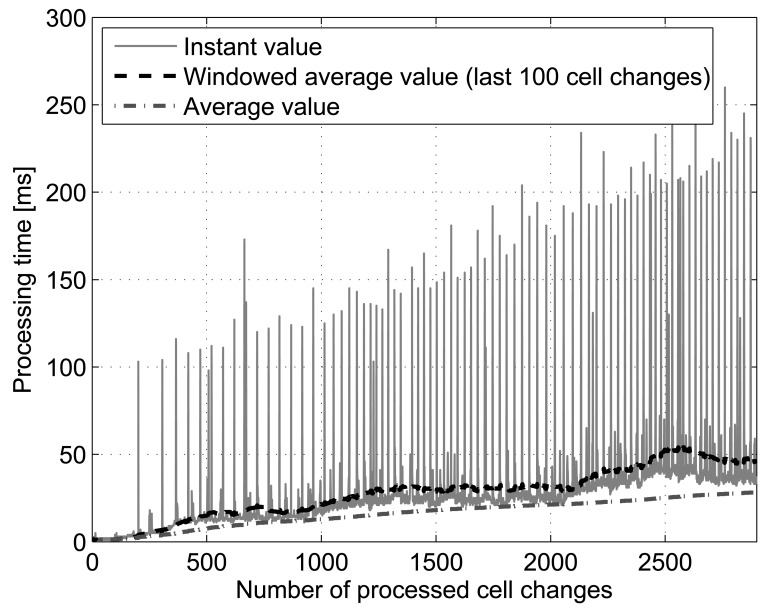
Processing time spent by a mobile phone for processing each new cell and estimating the most probable next location using Active LeZi and PPM without exclusion algorithm.

The previous figure gives just a glimpse of the differences between the processing time of the algorithms. However, it is also interesting to know the time it takes for a mobile phone to process each symbol (cell) as the time goes by and new cells are visited by the user. Figure 9 shows the time spent by a smartphone to process and generate a prediction for each new cell in every cell change in the 4 days trace. The smartphone used for the measurement is an HTC Hero with Android version 2.1-update 1. The test was driven with full battery, without connecting the terminal via USB to the computer, WiFi and GPS disabled, no SIM inserted, no other applications running and screen bright dimmed, in order to isolate the execution of the algorithms as much as possible. The 4 days trace was loaded into the device, and a simple application that takes one symbol (cell) at a time and executes the Active LeZi+PPM without exclusion algorithm was run over the entire trace. The algorithm selected represents the worst case in terms of resource consumption, since it uses the biggest tree and the probability calculation method that takes more time to estimate the next location, as shown before. In the figure we can see the instantaneous time spent for processing each new cell and estimate the next location, the running average of the process and the average made over the last 100 cell changes. Both averages smooth the peaks that can be seen at intervals that can be caused by external factors to the prediction process (e.g., operating system procedures, network operations…). As it can be seen, at the end of the trace, it takes around 50 milliseconds to make a prediction on the device.

**Figure 10.**
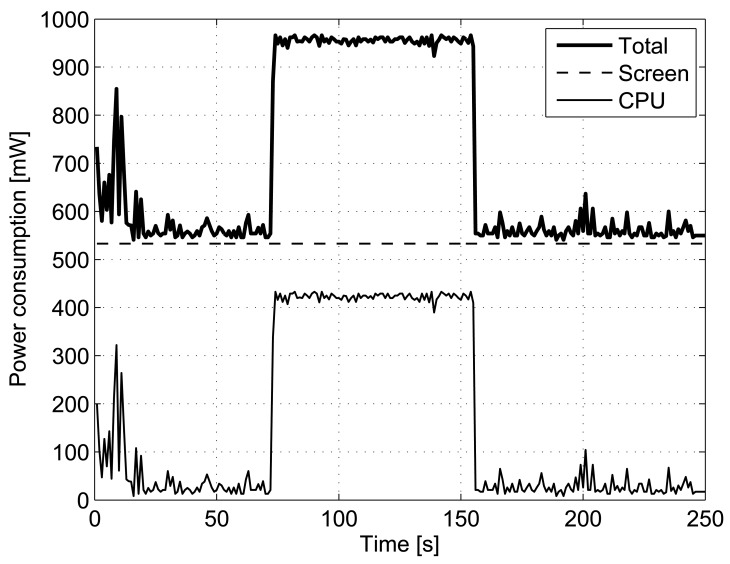
Power consumption of a mobile phone for processing each new cell and estimating the most probable next location using Active LeZi and PPM without exclusion algorithm.

Finally, the last resource to be analyzed is the power consumption derived from the algorithm execution in the terminal. In order to measure that, we have used the PowerTutor tool for Android phones (http://ziyang.eecs.umich.edu/projects/powertutor/). Using the same setup explained before, the 4 days trace was processed. Figure 10 shows the power consumption incurred during the period of execution of the prediction algorithm. Although the entire trace is processed continuously for this test, in the actual scenario this power consumption would be extended along the 4 days, *i.e.*, the algorithm is executed only when a new cell change happens, thus the power consumed by the CPU, which is around 400 mW, is bounded to the few miliseconds of processing for each cell change.

### 3.4. Using More Symbols

For some applications it could be useful not to know just the most probable next location, but the two or three most probable ones. Therefore we have measured hit rate considering a prediction error when the actual next location matches neither the most probable next location (1 symbol) nor the second most probable one (2 symbols) nor the third one (3 symbols).

**Figure 11.**
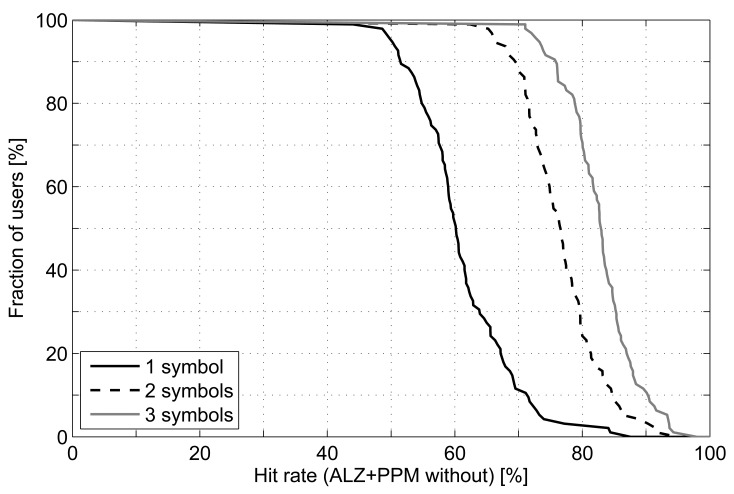
Hit rate attained by Active LeZi+PPM without exclusion when different number of symbols are used as prediction.

Figure 11 shows the results of applying this measurement over the predictions made with Active LeZi combined with PPM without exclusion. We can see a remarkable increase of hit rate when using two symbols, such that around 90% people attain at least a hit rate of 70%. The use of three symbols also improves the performance, but with less differences. An explanation for this result is that a trace gathers every cell change: the ones caused by user movement and those produced by detecting better signal from another base station when user is not moving. This last event is usually observed in traces as frequent changes between two or three cells, although this set of cells should be considered as an only one. Using more symbols as predictions is like clustering cells, and may be one of the reasons why hit rate improves so much.

## 4. Bus Lines Recommender System

In the previous section, several performance measurements were shown so as to evaluate the location prediction algorithms studied in this article as isolated entities. However, these predictors are thought to be working as part of pervasive or AmI applications. Therefore, it is worth to design an application that integrates them in one of this kind of systems in order to see how well they can work being part of a more complex application, which will be executed in a limited device such as a cell phone.

With this goal in mind, we have developed a prototype for Android cell phones that makes recommendations of bus lines based on the users' trajectory predicted by the algorithms. The general idea consists of monitoring the path followed by the user, combining this information with the prediction of the next location, and compare these data with the available bus lines, searching for those that best match with the potential path.

One of the advantages of this system is that the mobility tracking is made without using the GPS. Instead, the GSM approach explained in Section 2 has been followed to track users' mobility. The bus lines data could be accessed by making requests to some web service or REST API, but in this case we have saved the information into a database on the mobile phone itself in order to avoid Internet connections, which means energy consumption, third party dependence and extra response delay.

As explained before, each cell change triggers the prediction algorithms. They calculate the most probable next location in terms of another cell. This potential next location together with the path followed by the user is her trajectory. From this trajectory, just the last part is considered—the last part means the two most recently visited cells, the current one and the estimated future cell. This trajectory window is then compared against the bus lines, which have been previously mapped into sequences of cells and stored in the terminal memory.

Let Table 2 be an example of the set of bus stops from an example line, where we can see the identifiers of the BSs (cells) from which a terminal can receive signal in each bus stop.

Suppose a user whose usual path is *L* = *abcde fg*. At some point in time, user's phone will switch from cell *b* to *c*. At this point, the prediction algorithms will calculate the most probable next location and, as the usual path is the one shown above, the estimated next cell will be *d*. Therefore, the trajectory window considered will be {*a, b, c, d*}. Comparing this sequence with the set of bus lines, the application notices that the line represented in Table 2 matches with the potential trajectory of the user (see bold symbols in the table), thus resulting in a recommendation of this bus line.

The structure of the stored data of bus lines has an important advantage. The cell to which the terminal is connected to at some specific place may not be always the same one. Since BSs coverage zone are overlapping, it may be the case that walking the same path several times, the resulting sequences of cells are quite different. However, if this path is followed frequently by the user, the algorithms will learn the different sequences of symbols, predicting any of the cells covering that next location. Besides, since all the cells corresponding to a bus stop are stored and linked to that stop, no matter which BS the user connects to each time, there will always be a matching one in the corresponding bus stop.

As it can be realized with the previous example, there are many elements involved in the recommendation system: the bus lines information retrieval, mapping bus stops to their corresponding GSM cells, user motion tracking, location predictions and the orchestration of all the elements. The following subsections provide a glimpse of the architecture supporting the system as well as some of the tests made with the application.

### 4.1. Bus Line to Cell Sequence Translation

One of the main elements used by the recommendation system is the information related to the bus lines, *i.e.*, the identifier of each line, the bus stops belonging to each one (differentiating both directions) and the name and coordinates of those bus stops. Besides that, since we are using GSM-based location tracking, every trajectory needs to be translated into the corresponding sequence of cells identifiers.

In order to retrieve all the data related to the bus lines, we have used the information of the Local Transportation Enterprise (EMT) of Madrid. From its webpage (http://www.emt.com), we have extracted the data mentioned before by parsing the HTML code by means of an auxiliary Java application.

Having all these data, the next step is to map each bus stop with its corresponding cell or cells. In order to do that, we performed the following process. First, every pair of consecutive bus stops is processed so as to check if they are more than 200 m apart. If that is the case, a new fictitious bus stop is created in order to avoid long distances without any reference to be compared with respect to the user's trajectory. After having stored every bus stop, both real and fictitious ones, together with their coordinates, it is time to do the mapping. In order to do that, Google Gears Geolocation API was used for obtaining the coordinates of every BS. For each bus stop, all the BSs placed in a radius of 150 m are considered as potential BSs from which a terminal could receive signal from the bus stop.

**Figure 12.**
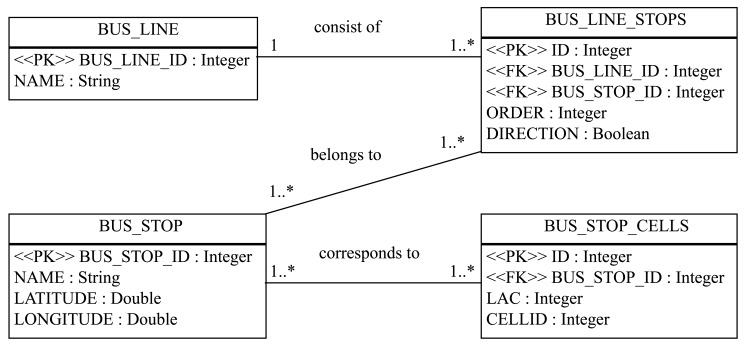
Data model corresponding to the database with the bus lines and corresponding cell identifiers information.

With all this information, we have created a database with the structure shown in Figure 12. This database is one of the basis of the application, which will be described next.

### 4.2. Application Architecture

**Figure 13.**
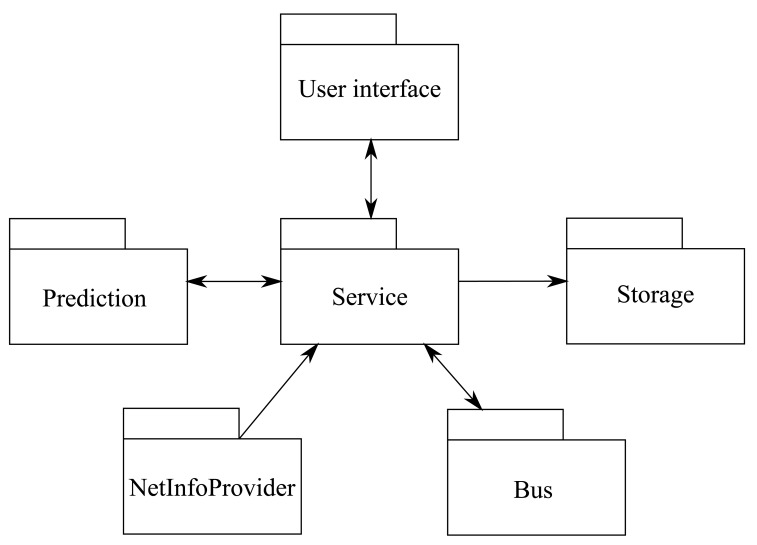
Block diagram of the recommender application.

In order to handle all the application functionality, we have divided it into independent modules. Figure 13 shows the block diagram of the recommender system, where each module is described below.

- NetInfoProvider. This is the block in charge of listening to cell changes, through the TelephonyManager API of Android. Each time it detects a cell change, the information of the new cell is gathered and sent to the central component.
- Storage. It is the component that deals with storage and retrieval of information: BS coordinates database and file read/write (for log purposes).
- Bus. This package is intended to manage the bus lines information: management of the database explained in the previous subsection and search of additional information (e.g., the time until a bus arrives at some stop).
- Prediction. All the logic related to the location prediction algorithms is encapsulated in this module: model building, tree management, prediction calculation…
- Service. This is the central unit of the application. It keeps a thread where the data of the new cell is received and processed, and another one to calculate the prediction. Once the trajectory window (last two visited cells, the current on and the estimated future one) is available, another thread is started to search in the bus lines database in order to search for matching bus lines. Finally, the results are shown through the user interface.
- User interface. This last component deals with the map and related issues that allow to show the path of the user, the data of the bus lines and the recommendations made by the system.

### 4.3. Testing the Application

**Figure 14.**
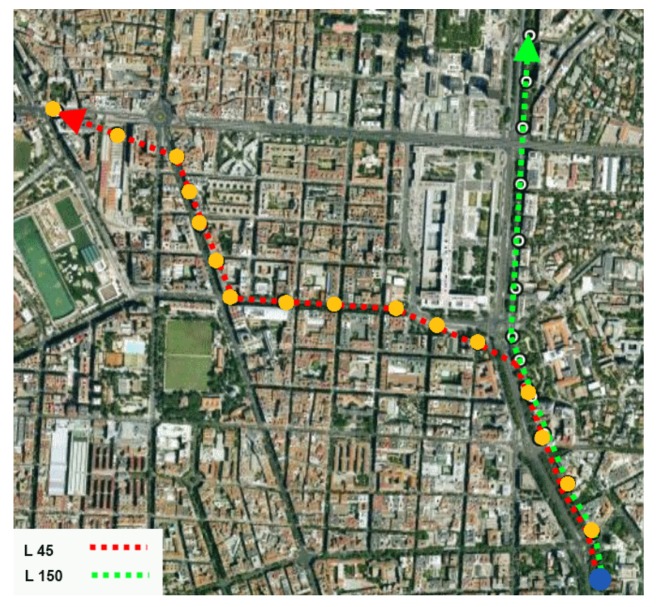
Test scenario. Paths marked in red and green are the bus lines considered. Blue point is the starting location of the user. Yellow dots are the places where the terminal switches from one cell to another and makes the prediction about the next location.

In order to check the recommender system, the following test was performed. The scenario shown in Figure 14 was built, which consists of two bus lines from EMT, lines 150 and 45. They were chosen because of two reasons:

- Both bus lines' trajectory has a common section, sharing several bus stops. Therefore, in such trajectory section the two lines will have the same sequence of cells. This feature will allow us to test the capability of the system of recommending both lines when they match the user's path.
- At some point of their trajectory, they take almost perpendicular directions. This fact will be useful in order to test two aspects: first, if the algorithms are able to predict the correct next location depending on which path is covered most frequently by the user; and second, if the application is able to recommend only the bus line that matches the path predicted by the algorithms.

In order to make the testing process easier, we have developed a module that simulates the movement of the user. This module triggers cell changes with the information contained in an external file, which includes the sequence of cells that represents the user's path.

Before testing the recommender system itself, we have simulated a user who covers the path of line 45 six times and the path of line 150 four times. This way the prediction algorithms learns the hypothetical mobility model of the user, supposing a user who usually takes line 45 route and sometimes changes this pattern and takes the path of line 150.

Once having the mobility models built and stored in the application, it is time to test the bus lines recommendation. For that, we started to simulate the movement of the user, from the point marked in blue in Figure 14. The user goes straight ahead along the principal street, changing from one cell to another in every yellow dot in the picture. At these points, a new prediction is made, where the estimated next location is the next yellow dot. During the first path section, the recommendation system suggests both lines, 45 and 150, since both of them matches with the predicted path of the user. When she reaches the bifurcation, the next prediction is on the path of line 45. The recommender system realizes this fact, and deletes line 150 from the suggestion.

## 5. Conclusions

Along this paper, we have evaluated three LZ family algorithms (LZ, LeZi Update and Active LeZi), by separating them into two independent phases and taking into account hit rate and resource consumption. The following conclusions can be drawn from this work: (i) Active LeZi updating scheme achieves the highest hit rate at the expense of being the highest memory consumer; (ii) the best probability calculation method depends on the updating scheme and the trace to be processed: PPM methods are usually the best ones, whereas Vitter method is much faster; (iii) for the best combination in terms of accuracy (ALZ+PPM without exclusion), half of the analyzed population achieves, at least, a hit rate of 60%, number that decreases very fast, having just around 5% of the population achieving a hit rate of, at least, 75%.

Regarding the integration of the predictors in a recommendation system executed in a mobile phone, we have checked its feasibility Besides, by simulating a user's movement, it has been shown that the recommendation process works as expected: it suggests all the bus lines whose trajectory matches the potential one of the user, discarding lines as the path of the user changes.

With respect to the analysis done in [11] we have considered an entire set of 95 anonymous users' as well as controlled traces we have recorded. As compared to Song's work [10], we have studied the algorithms as two independent phases, we have included Active LeZi algorithm, and used GSM-based traces, thus covering a countrywide area.

It would be interesting to consider Markov models in future works to check if order-2 Markov model achieves better results than LZ algorithms as shown in [10] (where a campus wide network was considered) when processing country wide location data. It would also be interesting to study, among others, the following topics: (i) how to include time information in the predictions to know also when the user will move; (ii) how to filter cell changes when user does not move; and (iii) to study the energy consumption associated to the execution of the algorithms on the terminals.

## Figures and Tables

**Table 1.** Frequency of substrings following the current context for ALZ tree.

***l*_2_ = *ab***	***l*_1_**= *b*		***l*_0_ = *γ***				
c:l	a:l	da:l	a:l	ba:l	bda:l	cd: 1	dab:l
esc:2	c:0	esc:0	ab:2	bc:0	c:0	cdc:l	dc:0
	cd:l		abc:l	bcd:l	cb:0	d:0	dcb:l
	d:0		b:3	bd:0	cbd:l	da:0	esc:0

**Table 2.** Example of the mapping from the bus line (sequence of bus stops) to the cells covering each bus stop.

**Bus stop ID**	**Cells ID**			
1	**a**	*o*		
2	*a*	**b**	*p*	
3	*b*	**c**	*r*	
4	**d**	*e*	*f*	*t*
5	*e*	*f*	*g*	*u*
6	*h*	*i*	*m*	
7	*j*	*l*		
